# Glances at the Insane in Other Lands

**Published:** 1888-12-15

**Authors:** 


### GLANCES AT THE INSANE IN OTHER LANDS.

By a Medical Superintendent.

*(Continued from page 134.)*

### II.—NEW ZEALAND.

In the entire Colony of New Zealand there are 1,695 registered insane persons, showing an increase of 83 during the year. Excluding the Maories, this gives a proportion of one lunatic to every 360 sane people. In England the proportion is 1 to 348, and in Scotland 1 to 420.

Dr. MacGregor, the author of the report under notice, bewails the fact that the asylums are becoming overburdened with old and helpless cases, and he says the medical superintendents find themselves in the predicament of either turning out these poor old friendless creatures, or allowing them to occupy beds which are needed for acute and curable cases; but inasmuch as the highest number of lunatics in any district in New Zealand would seem to be only 500, the proper remedy is assuredly to enlarge the existing asylums. The presence of these demented cases would be in no way injurious. In fact, they would merely, by their numbers, permit of proper classification and separation of the various classes of the insane, and so they would prove beneficial rather than otherwise. It has been shown over and over again that the presence of these imbeciles in no way interferes with the treatment of acute cases—at least, when the total number of inmates is under 800, or even 1,000. Instead of building one central hospital for curable cases, which would be hardly possible in New Zealand, Dr. MacGregor recommends the erection of one central building for imbeciles and idiots. It is extremely doubtful whether the colony will need this for the next hundred years, and in the meantime it could hardly fail to be an additional source of expense, for although the weekly cost of maintenance might be a trifle lower than in the ordinary asylums, it is certain that with the withdrawal of the imbeciles from these institutions their weekly rate would rise, as the establishment charges would be distributed over a smaller number. It is an interesting thing to notice that the New Zealand asylums are curing more patients under the old system of asylum management than New South Wales is doing with a Central Hospital for acute cases. In New South Wales the percentage of recoveries calculated on the admissions was 41·26, while in New Zealand it was 43·61.

The percentage of deaths is the lowest we have yet met with, being at the rate of 6·13 on the average number resident. The total number of deaths was 101. Ten of these were from consumption, and 41 from Brain disease in its Protean forms. In looking over the table indicating the causes of insanity, we find the same old story told in the same old language. Drink heads the list with 39 out of a total of 415 admissions, and hereditary influence comes next with 28; but in 130 the cause of the disease could not be ascertained, so that the numbers quoted would doubtless have to be largely increased could we get satisfactory information.

What a strange medley of nationalities some of the New Zealand asylums present! We read of English, Scotch, Irish, New Zealanders, Australians, French, Germans, Norwegians, Swedes, Danes, Italians, Chinese, and Maories, and as if even that long list were not enough 69 are put down as belonging to "other countries." English medical superintendents say that Scotch men and Irish women are almost invariably troublesome elements in English asylums, but what would they think of such a conglomeration as we have noticed above. New Zealand medical superintendents are much to be pitied.

The average weekly cost per head is about 10s.

In the report of the Auckland Asylum we note reference made to a great amount of overcrowding, and to relieve this some enlargement of the building has been carried out, during which great difficulties were experienced in maintaining discipline. Typhoid fever broke out, but apparently its progress was soon arrested. The workshops were burned down. Altogether, the medical superintendent must have had a trying time of it, and we hope he has had a happy issue out of all his troubles. There were 363 patients in the asylum.

The Seacliff Asylum, Dunedin, contains 501 patients. It would seem that 165 male patients were employed on the farm, 109 at indoor work, and nine in the shops. Only 36 men and 86 women were idle.

At Sunnyside there are 346 patients. The asylum is superintended by Dr. Levinge, and the Inspector gives him a high meed of praise for the condition to which the asylum has been raised.

In the Wellington Asylum are 123 males and 89 females. It would seem that all the male patients, except 20, are employed at some kind of work, and of the women only 19 are idle. Doubtless much of the work done is not very profitable, but we cannot help thinking that so high a percentage of workers in this and in the other asylums spoken of is highly creditable to our Colonial confréres.

The Asylum at Nelson is quite a small one, containing only 100 patients.

The Hokitika Asylum is similar as regards numbers. There is nothing in the report of either calling for special mention.

